# Phenolic Profiles and Antioxidant Activity of Germinated Legumes

**DOI:** 10.3390/foods5020027

**Published:** 2016-04-09

**Authors:** Do Tan Khang, Tran Nhan Dung, Abdelnaser Abdelghany Elzaawely, Tran Dang Xuan

**Affiliations:** 1 Graduate School for International Development and Cooperation, Hiroshima University, 1-5-1 Kagamiyama, Higashi-Hiroshima, Hiroshima 739-8529, Japan; dtkhang@ctu.edu.vn; 2 Biotechnology Research and Development Institute, Can Tho University, Vietnam; tndung@ctu.edu.vn; 3 Department of Botany, Faculty of Agriculture, Tanta University, Tanta 31527, Egypt; elzaawely@agr.tanta.edu.eg

**Keywords:** antioxidant activity, DPPH•, germination, legumes, Phenolics, reducing power

## Abstract

Bioactive compounds, which are naturally produced in plants, have been concerned with the food and pharmaceutical industries because of the pharmacological effects on humans. In this study, the individual phenolics of six legumes during germination and antioxidant capacity from sprout extracts were determined. It was found that the phenolic content significantly increased during germination in all legumes. Peanuts showed the strongest antioxidant capacity in both the DPPH• (1,1-diphenyl-2-picrylhydrazyl) method and the reducing power assay (32.51% and 84.48%, respectively). A total of 13 phenolic acids were detected and quantified. There were 11 phenolic constituents identified in adzuki beans; 10 in soybeans; 9 in black beans, mung beans, and white cowpeas; and 7 compounds in peanuts. Sinapic acid and cinnamic acid were detected in all six legume sprouts, and their quantities in germinated peanuts were the highest (247.9 µg·g^−1^ and 62.9 µg·g^−1^, respectively). The study reveals that, among the investigated legumes, germinated peanuts and soybeans obtained maximum phenolics and antioxidant capacity.

## 1. Introduction

Consumption of legumes potentially reduces the risk of chronic diseases [1] such as stroke, type II diabetes [2], cardiovascular [3], and gastrointestinal cancer [4]. In addition to fiber, legume grains also contain many substances to improve health such as vitamins, minerals, and other substances, including phenolic compounds [2]. Phenolic compounds are resistant to oxidation and protect cell damage to prevent the risk of degenerative diseases thanks to antioxidative, anti-inflammatory, antiallergic and anticarcinogenic activities [5,6]. Various studies have proved the dramatic change in beneficial substances that are heavily linked to germination of grains. In this process, endoenzymes are activated to hydrolyse macro molecules including starches, proteins and lipids to produce nutritional elements of a plant’s development [7].

Total phenolics and antioxidant activities of grains and germinated grains have been widely investigated. Germination produced high phenolic content and consequently increased the antioxidant activities of lupin seeds (*Lupinus angustifolius* L., *cv*. Zapaton) [8]. Xu *et al.* [6] reported that free phenolics and avenanthramides, the major phenolic component of oats, increased during the germination of oats. Lin and Lai [9] obtained similar results when they compared the phenolic content and reducing powers of 19 domestic legumes in Taiwan, including soybeans, black soybeans, adzuki beans, and mung beans. However, the identification of individual phenolics and the relationship among phenolic components in germinated legumes are rarely reported. The aim of this study was to investigate the changes in phenolics and antioxidant capacity of six popular legumes during germination to evaluate their potential uses in foods and pharmaceuticals.

## 2. Results

### 2.1. Total Phenolic Content

The change in total phenolic content in legumes during germination is illustrated in Table 1. The phenolic concentration dramatically increased (*p* < 0.05) in all legumes after five days of germination. In ungerminated grains, the content of total phenolics ranged from 5.80 (mung beans) to 18.21 (peanuts) mg·GAE·g^−1^ dry sample. After 5 days of sprouting, the phenolic content in mung beans, white cowpeas, soybeans and peanuts increased 2-fold, while that in black beans and adzuki beans showed about a 50% and 25% increase, respectively. In particular, peanuts had the highest concentration, which was 37.59 mg·GAE·g^−1^, followed by soybeans (28.27 mg·GAE·g^−1^), white cowpeas (19.46 mg·GAE·g^−1^), adzuki beans (16.96 mg·GAE·g^−1^), black beans (16.47 mg·GAE·g^−1^), and mung beans (14.97 mg·GAE·g^−1^). 

### 2.2. Identification and Quantification of Phenolic Components

**Figure 1:**
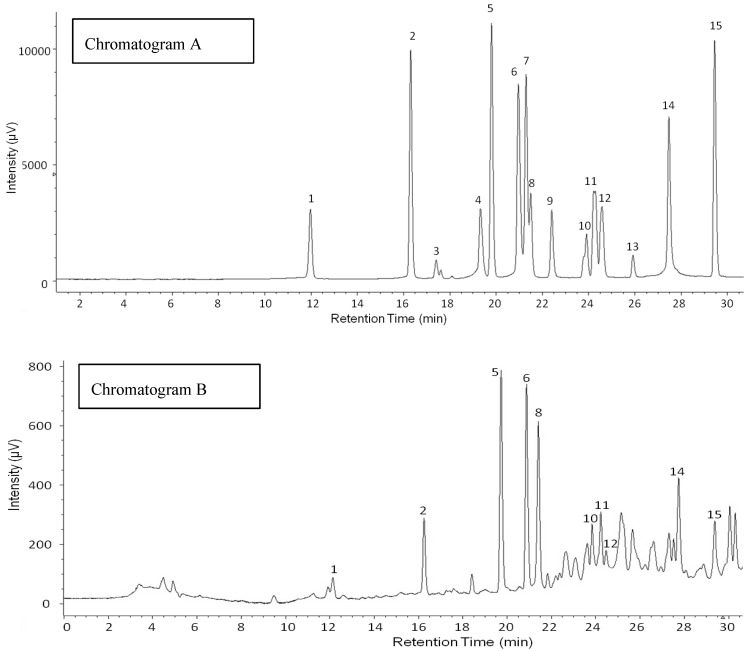
High Performance Liquid Chromatography (HPLC) chromatograms of phenolic standards (**A**) and soybean sprouts (**B**); 1: gallic acid; 2: protocatechuic acid; 3: catechol; 4: chlorogenic acid; 5: *p*-hydroxybenzoic acid; 6: vanillic acid; 7: caffeic acid; 8: syringic acid; 9: vanillin; 10: ferulic acid; 11: sinapic acid; 12: *p*-coumaric acid; 13: benzoic acid; 14: ellagic acid; 15: cinnamic acid.

Phenolic profiles of legume sprouts are shown in Table 2. Different concentrations of 13 out of 15 standard phenolics (Figure 1) ranged from 2.2 µg·g^−1^ to 636.0 µg·g^−1^, consisting of protocatechuic acid, *p*-hydroxybenzoic acid, vanillic acid, caffeic acid, syringic acid, vanillin, ferulic acid, sinapic acid, *p*-coumaric acid, benzoic acid, ellagic acid and cinnamic acid, and were identified in six legume extracts. There were 11 compounds found in adzuki beans, 10 in soybeans, 9 in black beans, mung beans and white cowpeas, and 7 in peanuts. Sinapic acid and cinnamic acid were detected in all legume sprouts. Germinated peanuts contained the highest concentrations in the two phenolic acids, which constituted 247.9 µg·g^−1^ and 62.9 µg·g^−1^, respectively. Sinapic acid was also the dominant compound in adzuki bean sprouts with 150.7 µg·g^−1^. Benzoic acid was the most abundant in germinated mung beans (636.0 µg·g^−1^), black beans (253.5 µg·g^−1^), white cowpeas (199.5 µg·g^−1^), and adzuki beans (124.8 µg·g^−1^), but it was not found in peanut and soybean sprouts, which in contrast contained the highest concentrations of ferulic acid (330.0 µg·g^−1^) and syringic acid (327.5 µg·g^−1^).

### 2.3. Antioxidant Capacity of Germinated Legume Extracts

The antioxidant activities of germinated legumes were evaluated by the DPPH**•** method and the reducing power assay, as shown in Table 3. It was found that the six legume extracts had different antioxidant activity levels at a 0.1 mg·mL^−1^ dose. In particular, peanuts had the highest antioxidant activity at 32.51%, which was significantly different from the others (*p* < 0.05), while black beans showed the lowest antioxidant activity (7.44%). The reducing power of germinated peanuts obtained maximum activity (84.48%) compared to other studied legumes (Table 3), whereas the lowest antioxidant activity was mung beans (26.45%). Although adzuki beans had higher DPPH**•** scavenging activity than black beans and white cowpeas, the reducing power capacity of adzuki beans was, in contrast, significantly lower than that of black beans and white cowpeas (Table 3).

### 2.4. Correlation between Phenolics and Antioxidant Activity

**Figure 2:**
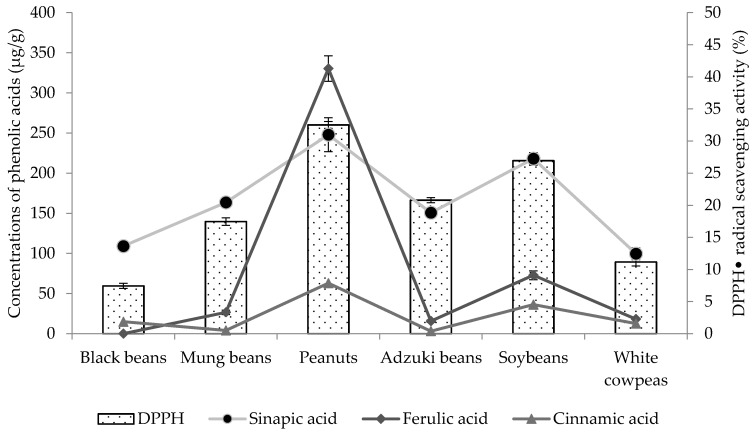
Correlation between 1,1-diphenyl-1-picrylhydrazyl (DPPH•) radical scavenging activity and concentrations of detected phenolic acids.

**Figure 3:**
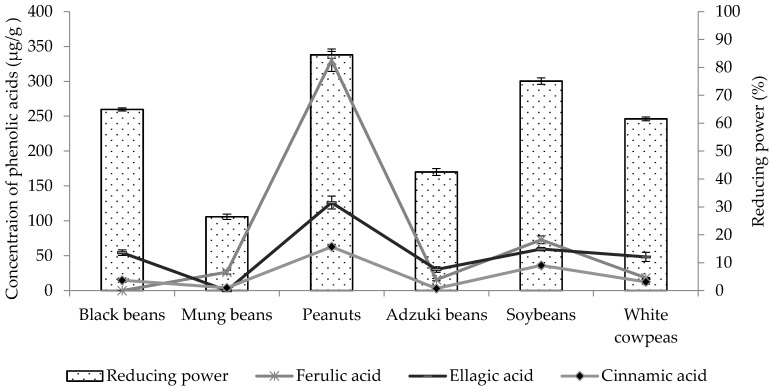
Correlation between reducing power and concentrations of detected phenolic acids.

It was observed that the levels of antioxidant activity depend on both concentrations and types of phenolics, and the correlations between antioxidant activities and phenolics are shown in Table 4, Figure 2 and Figure 3. Regarding DPPH**•** radical scavenging activity, sinapic acid, ferulic acid and cinnamic acid showed the strongest correlations with the R^2^ values of 0.965, 0.789, and 0.744, respectively. Ellagic acid, cinnamic acid and ferulic acid showed maximum correlations with reducing power, as the *R*^2^ values were 0.910, 0.848 and 0.625, respectively (Table 4).

## 3. Discussion

### 3.1. Total Phenolic Content

Total phenolics are naturally produced during the growth and development of plants to protect themselves from biotic stresses such as diseases, insects and environmental stresses [5,10]. The dramatic change in phytochemicals in the germination process has been considered as a natural phenomenon of plants. This research supports the theory that phenolic content positively increases with germination, as shown by the six legumes. The rise in polyphenol content after germination has been broadly reported in chickpeas [11], lupin seeds [12], beans, lentils and peas [13], oats [6], and *Ceiba pentandra* seeds [14]. Because the metabolism initiates in the presence of water, the particles inside the grains change, and accordingly generate a great amount of energy and new compounds, including phenolics [6,13]. During the germination of peanuts, resveratrol, a typical phenolic in peanuts, was highly synthesized [15]. In addition, flavonoids and epicatechins were other two dominant phenolics in this legume [16]. In soybeans, the primary phenolics were flavonoids, while those in mung beans, adzuki beans, black beans and white cowpeas were phenolic acids [17]. Some studies have shown that levels of phenolics depend on extracting solvents. According to Nepote *et al.* [18], methanol was ideal for extracting resveratrol and stilbenes. For phenolic acids, to achieve the high levels of extraction, ethanoic acids are solvents that are commonly used [17]. For these reasons, in this study, the total phenolic content of peanuts was reasonably higher than that of other legumes.

### 3.2. Identification of Phenolic Profiles in Germinated Legumes

Phenolic compounds consist of phenolic acids, tannins, and flavonoids, which are generally produced in plants, and the abundance of these chemicals depends on species and stage of growth [6,13,19]. Sinapic acid and cinnamic acid are common phenolics found in plants [20], so these could be the dominant phenolics in all six legumes. Sebei *et al.* [21] determined eight phenolic compounds in peanut kernels including caffeic acid, dihydroxyphenylacetic acid, syringic acid, *p*-coumaric acid, rutintrihydrate, nephtoresorinol, *trans*-2-dihydroxycinamic acid and dihydratequercetin. In addition, *p*-hydroxybenzoic acid and chlorogenic acid were also detected in both peanut kernels and skin [15]. In this study, ferulic acid, sinapic acid and ellagic acid highly contributed to the total phenolic content of peanuts because these phenolic acids had the highest concentrations. Adzuki beans and black beans are dark skin seeds that contain anthocyanidin pigments consisting of delphinidin, cyanidin, pelargonidin, malvidin, and petunidin [22]. According to Fidrianny *et al.* [23], the sprout extracts from soybeans showed higher flavonoid content than those from white cowpeas and mung bean. The main phenolic compounds in soybeans were isoflavones, which were identified as aglycones (daidzein, genistein, glycitein), glucosides (daidzin, genistin, glycitin), malonylglucosides, (malonyldaidzin, malonylgenistin, malonylglycitin), and acetyl glucosides (acetyl daidzin, acetyl genistin, acetyl glycitin) [9].

### 3.3. The Antioxidant Capacity of Germinated Legume Extracts

Antioxidant activity is closely related to phenolic content [6,24,25]. In this study, peanuts contained a maximum concentration of phenolics, which may result in the strongest antioxidant activity of the legume. Furthermore, according to Corral-Aguayo *et al.* [26], when compared to the antioxidant activity, resveratrol had stronger activity than flavonoids. In addition, the main substances in soybean phenolics were flavonoids, whereas, in other legumes (chickpeas and black, red, and white cowpeas), the major components were phenolic acids [17]. This may explain why soybeans had greater antioxidant activities than the other four legumes in the DPPH**•** assay.

### 3.4. Correlation between Phenolics and Antioxidant Activities

It is believed that phenolic structures play a crucial role in bioactive activities [23]. Particularly, the number and location of hydroxyl groups in phenolic structures inextricably link to antioxidant activity [27]. If an additional hydroxyl group is added to *ortho* or *para* positions of the benzen ring, the antioxidant activity will significantly increase. Principally, the *ortho* position can create the intramolecular hydrogen bond, so such a position is proposed to be more effective than a *para* or a *meta* one [28]. Similarly, the antioxidant capacity is higher if there is one or more methoxy groups introduced. Therefore, the inhibiting effectiveness of cinnamic acid derivatives was stronger than that of benzoic acid derivatives [29]. As a result, sinapic acid was the most correlative with the antioxidant activity of legume phenolics because sinapic acid contains both 3,5-dimethoxyl and 4-hydroxyl groups [30]. Sinapic acid was found in various plants including rye, fruits and vegetables, and was especially abundant in rapeseeds (over 73% rapeseed phenolics). Moreover, biological activities such as anxiolytic, anti-inflammatory and peroxinitrite scavenging were successfully evaluated [30]. Ellagic acid is highly contained in berries (raspberries and strawberries), walnuts, tea, red wine, longan seed, mango, kernel, pomegranate, and herbal plants [31,32]. Ellagic acid has positive effects on anti-inflammation, anti-proliferation, anti-angiogenesis, anticarcinogenesis, antimutagenensis, anti-cancer, and antiradical [32]. In addition, the high antioxidant activities of ellagic acid consisting of ferric thiocyanate, hydrogen peroxide scavenging, DPPH**•** scavenging, 2,2′-azino-bis (3-ethylbenzthiazoline-6-sulfonic acid) (ABTS) radical scavenging activity and superoxide anion radical scavenging, ferrous ions (Fe^2+^) chelating activity, and ferric ions (Fe^3+^) reducing ability were confirmed [31].

## 4. Materials and Methods

### 4.1. Samples

Commercial legume grains, including mung beans (*Vigna radiata*), white cowpeas (*Vigna unguiculata*), black beans (*Vigna cylindrica*), adzuki beans (*Vigna angularis*), soybeans (*Glycine max*) and peanuts (*Arachis hypogaea*), were purchased from the Coopmark supermarket, Can Tho city, Vietnam. Seeds were germinated at 30 °C for five days, and then milled into powder. The powdered samples were kept in plastic bags and stored at 4 °C for further analysis.

### 4.2. Phenolic Extraction

A quantity of 5 g of grain powder was homogenized in 50 mL of 80% methanol, stirred continuously for 24 h at room temperature. The mixture was then separated by centrifuging at 5000 rpm (3000× *g*) for 15 min. The supernatant was taken and filtered through the filter membrane with a size of 0.45 μm. The pellet was extracted twice, and the solutions were mixed together. The total extracts were dried using a rotary evaporator at 30 °C. Methanol was used to dissolve the dried extracts.

### 4.3. Total Phenolic Content

The total phenolics were determined by the Folin-Ciocalteu method described by Taga *et al.* [33] with several modifications. A volume of 100 µL of diluted sample (1 mg·mL^−1^) was mixed with 100 µL of 80% methanol. Then, an aliquot of 2 mL of Na_2_CO_3_ 2% was added and left for 2 min. Next, 100 µL of a Folin-Ciocalteu reagent was added. The absorbance was measured at 750 nm after 30 min using a spectrophotometer (DU 640 (Beckman Coulter, Germany)). Total phenolic content was estimated based on the gallic acid standard curve (0, 20, 40, 60, 80, and 100 µg·mL^−1^) and expressed as mg gallic acid equivalents (GAE) g^−1^ dry matter.

The 5-day germinated legumes were taken for evaluating the antioxidant activity and phenolic identification.

### 4.4. Evaluation of Antioxidant Activity

#### 4.4.1. Reducing Power Assay

For the assay of reducing power, the protocol of Singhal *et al.* [34] was used and described as follows. One milliliter of the filtrate was mixed with 2.5 mL of phosphate buffer (pH 6.6) and 2.5 mL of K_3_[Fe(CN)_6_] (1%), which was followed by incubation at 50 °C for 20 min. The reaction was then stopped by adding 2.5 mL of trichloroacetic acid (10%), followed by centrifuging at 3000 rpm (1000× *g*) for 10 min. The supernatant (2.5 mL) was mixed with distilled water (2.5 mL) and 5 mL of FeCl_3_ solution (1%), and the absorbance was measured at 700 nm. In the reducing power assay, the more the absorbance of the reaction increased, the more reducing power was obtained. The percentage of the reducing power was calculated based on the following formula: reducing power = [(Abs_extract_ − Abs_blank_)/Abs_blank_] × 100%, where Abs_extract_ is absorbance of extracts, and Abs_blank_ is absorbance of water.

#### 4.4.2. DPPH Radical Scavenging Assay

The radical scavenging capacity of the samples was tested based on the procedure described by Siddiqua *et al.* [35]. Briefly, the reaction contained 1 mL of extracts, 3 mL of methanol, and 150 µL of DPPH**•** 0.1%. The absorbance was recorded at 517 nm after 30 min. The capacity of radical scavenging was calculated with the following formula: %DPPH**•**_scavenging_ = [(Abs_control_ - Abs_ample_)/Abs_control_] × 100%, where Abs_sample_ is absorbance of extract solution, and Abs_control_ is absorbance of methanol in DPPH**•**.

### 4.5. Quantification of Phenolic Components in Germinated Legumes

Phenolic compounds found in plants usually exist in conjugated forms with sugars of esters. Therefore, to determine the phenolics in germinated legumes, an acidic fraction of the samples was obtained via the extraction method described in Xuan *et al.* [36]. The phenolic extracts were injected into a Jasco HPLC apparatus (Japan) consisting of a PU-2089 Plus pump, a LC-Net/ADC controller, and a UV-2075 Plus detector (set at 254 nm wavelength). A J-Pak Symphonia C18 column (5 µm × 4.6 mm × 250 mm) was used for separation. The column temperature was 25 °C. The two solvents used were absolute methanol (A) and 0.1% acetic acid (B). The gradient elution process began with the mobile phase A increasing from 5%–10% in the first 5 min with a flow rate of 1 mL·min^−1^, then increased from 10% to 90% for the next 45 min, and the last 10 min was 100% A. The peaks of the samples were identified and calculated based on the retention times and peak areas of the phenolic standards.

### 4.6. Statistical Analysis

All data were statistically analyzed using ANOVA in the Minitab 16.0 software (Minitab Inc., State College, PA, USA). Significant differences among the means were compared using Fisher’s test method with a confidential level of 95%. All experiments were carried out in triplicate and expressed as mean ± standard deviation (SD).

## 5. Conclusions

In this study, 13 phenolic compounds were detected in germinated grains of black beans, mung beans, soybeans, peanuts, adzuki beans and white cowpeas with high concentrations. Among these identified phenolic acids, sinapic acid, ellagic acid, ferulic acid and cinnamic acid showed high correlations with antioxidant activities. The findings showed that germinated legumes are a promising source of phenolic compounds with beneficial effects for human health.

## Figures and Tables

**Table 1:** Total phenolic content (mg·GAE·g^−1^ dry weight) of ungerminated and germinated legumes.

	Black Beans	Mung Beans	Peanuts	Adzuki Beans	Soybeans	White Cowpeas
0 h	11.74 ± 0.07 ^t^	5.80 ± 0.05 ^w^	18.21 ± 0.15 ^k^	12.21 ± 0.06 ^s^	12.12 ± 0.09 ^s^	7.79 ± 0.02 ^v^
24 h	13.17 ± 0.10 ^r^	11.47 ± 0.09 ^tu^	26.89 ± 0.11 ^f^	13.64 ± 0.17 ^q^	21.46 ± 0.05 ^i^	11.33 ± 0.09 ^u^
48 h	13.62 ± 0.07 ^q^	12.32 ± 0.36 ^s^	28.71 ± 0.16 ^d^	15.59 ± 0.05 ^n^	21.61 ± 0.11 ^i^	14.06 ± 0.08 ^p^
72 h	14.20 ± 0.18 ^p^	12.28 ± 0.13 ^s^	29.39 ± 0.04 ^c^	15.89 ± 0.05 ^n^	23.92 ± 0.08 ^h^	14.71 ± 0.07 °
96 h	13.47 ± 1.17 ^qr^	12.47 ± 0.09 ^s^	30.34 ± 0.09 ^b^	16.57 ± 0.05 ^m^	24.75 ± 0.15 ^g^	16.29 ± 0.11 ^m^
120 h	16.47 ± 0.14 ^m^	14.97 ± 0.09 °	37.59 ± 0.18 ^a^	16.96 ± 0.06 ^l^	28.27 ± 0.11 ^e^	19.46 ± 0.09 ^j^

Means ± SD (standard deviation) that do not share a superscript letter are significantly different at 95% confidential level.

**Table 2:** Phenolic components and concentrations (µg·g^−1^ dry weight) of legumes after 5-day germination.

	Black Beans	Mung Beans	Peanuts	Adzuki Beans	Soybeans	White Cowpeas
Gallic acid	nd	nd	nd	nd	21.3 ± 2.9	nd
Protocatechuic acid	nd	nd	15.1 ± 2.5 ^b^	2.2 ± 0.1 ^c^	41.5 ± 6.6 ^a^	nd
*p*-hydroxybenzoic acid	24.1 ± 1.0 ^b^	15.9 ± 0.3 ^b^	16.8 ± 0.1 ^b^	19.5 ± 0.6 ^b^	66.6 ± 0.8 ^a^	nd
Vanillic acid	43.8 ± 0.8 ^c^	5.2 ± 0.7^e^	67.6 ± 0.8 ^b^	25.2 ± 0.9 ^d^	189.2 ± 10.1 ^a^	nd
Caffeic acid	nd	nd	nd	nd	nd	70.8 ± 6.6
Syringic acid	79.8 ± 1.7 ^b^	16.7 ± 1.8 ^c^	nd	23.8 ± 0.8 ^c^	327.5 ± 14.9 ^a^	30.4 ± 5.3 ^c^
Vanillin	12.4 ± 0.8 ^b^	14.2 ± 0.5 ^b^	nd	15.9 ± 2.9 ^b^	nd	28.9 ± 3.3 ^a^
Ferulic acid	nd	26.6 ± 2.8 ^c^	330.3 ± 16.0 ^a^	15.7 ± 1.6 ^c^	72.9 ± 5.6 ^b^	18.2 ± 0.8 ^c^
Sinapic acid	109.1 ± 0.5 ^c^	163.5 ± 3.8 ^b^	247.9 ± 21.1 ^a^	150.7 ± 4.6 ^b^	218.1 ± 7.5 ^a^	99.65 ± 7.4 ^c^
*p*-coumaric acid	72.1 ± 3.1 ^b^	288.7 ± 3.6 ^a^	nd	14.4 ± 2.8 ^c^	18.1 ± 4.5 ^c^	81.95 ± 0.65 ^b^
Benzoic acid	253.5 ± 26.7 ^b^	636.0 ± 2.4 ^a^	nd	124.8 ± 51.9 ^c^	nd	199.5 ± 36.4 ^bc^
Ellagic acid	54.4 ± 3.9 ^ns^	nd	126.2 ± 89.3 ^ns^	30.3 ± 4.0 ^ns^	59.6 ± 2.4 ^ns^	48.3 ± 6.5 ^ns^
Cinnamic acid	14.85 ± 2.9 ^ab^	4.0±0.1 ^b^	62.9 ± 35.6 ^a^	3.1 ± 0.2 ^b^	36.2 ± 1.5 ^ab^	12.5 ± 3.2 ^ab^

Means ± SD (standard deviation) that do not share a letter in the same row are significantly different at 95% confidential level. ns: not significantly different; nd: not detected.

**Table 3:** Antioxidant activities of 5-day germinated legume extracts.

Sample	DPPH• Scavenging (%)	Reducing Power (%)
Black beans	7.44 ± 0.39 ^f^	64.92 ± 0.57 ^c^
Mung beans	17.46 ± 0.60 ^d^	26.45 ± 0.95 ^f^
Peanuts	32.51 ± 0.54 ^a^	84.48 ± 1.24 ^a^
Adzuki beans	20.80 ± 0.39 ^c^	42.51 ± 1.24 ^e^
Soybeans	26.94 ± 0.71 ^b^	75.08 ± 1.18 ^b^
White cowpeas	11.17 ± 0.63 ^e^	61.53 ± 0.68 ^d^

Means ± SD that do not share a superscript letter are significantly different at 95% confidential level.

**Table 4:** Linear correlation coefficient (*R*^2^) of antioxidant activities and individual phenolic acids.

Phenolic	DPPH Radical Scavenging	Reducing Power
Benzoic acid	−0.513	−0.831 *
Cinnamic acid	0.744	0.848 *
Ellagic acid	0.558	0.910 *
Ferulic acid	0.789	0.625
*p*-coumaric acid	−0.380	−0.763
*p*-hydroxybenzoic acid	0.407	0.325
Protocatechuic acid	0.657	0.568
Sinapic acid	0.965 **	0.439
Syringic acid	0.217	0.362
Vanillic acid	0.549	0.585
Vanillin	−0.760	−0.505

Gallic acid and caffeic acid were not included. Means with * and ** are significantly different at *p* < 0.05 and 0.01, respectively.

## Abbreviations

The following abbreviations are used in this manuscript:

Abs: Absorbance
DPPH•: 1,1-diphenyl-1-picrylhydrazyl
HPLC: High Performance Liquid Chromatography
GAE: Gallic Acid equivalents
SD: Standard Deviation
